# Global research trends and hotspots in erysipelas: a bibliometric analysis from 2000 to 2024

**DOI:** 10.3389/fmed.2025.1530278

**Published:** 2025-05-07

**Authors:** Chaoxi Zhou, Guangrong Yu, Qinglei Wang

**Affiliations:** ^1^Department of Emergency Surgery, Beijing Geriatric Hospital, Beijing, China; ^2^Department of Orthopedics, Beijing Geriatric Hospital, Beijing, China

**Keywords:** erysipelas, risk factors, bibliometric analysis, diagnosis and differential diagnosis, antibiotic resistance

## Abstract

**Background:**

Erysipelas, an acute bacterial infection affecting the dermis and lymphatic system, poses significant clinical challenges due to recurrence, antibiotic resistance, and diagnostic overlap with other skin infections. Despite growing research in this area, a comprehensive bibliometric analysis is lacking, leaving gaps in understanding the publication trends, key research hotspots, and collaborative networks that shape the field.

**Methods:**

This study conducted a bibliometric analysis of erysipelas research from 2000 to 2024 using the Science Citation Index Expanded (SCI-EXPANDED) within the Web of Science Core Collection. English-language articles and reviews were selected, and data were analyzed using VOSviewer, Bibliometrix, and CiteSpace to visualize publication volume, co-authorship networks, geographic distribution, keyword trends, and collaborations.

**Results:**

The results indicate a steady growth in erysipelas research from 2000 to 2024, with annual publication volumes increasing from 9 articles in 2000 to 30 articles in 2022, reflecting a significant rise in interest within the field. The United States leads in contributions with 69 publications and 2,739 citations, institutional analysis highlights Radboud University, Karolinska Institute, and others as key contributors in collaboration and citation impact. Keyword co-occurrence analysis identifies major research hotspots, including familial Mediterranean fever, lymphedema therapy, and the management of complex skin infections, with emerging themes like “liposuction” and “skin and soft tissue infections” gaining attention in recent years.

**Conclusion:**

This study highlights the growing advancements in erysipelas research, including insights into risk factors, diagnostics, and therapies, while emphasizing the need for continued collaboration and innovation to address challenges like antibiotic resistance, recurrence, and accurate differentiation from similar conditions.

## 1 Introduction

Erysipelas is an acute, localized bacterial infection affecting the upper dermis and lymphatic system, predominantly caused by *Streptococcus pyogenes* (1). Characterized by its rapid onset, erysipelas presents clinically with distinctive raised red patches and inflammation, often accompanied by systemic symptoms such as fever and chills (2). This condition is frequently recurrent and poses significant healthcare challenges (3, 4), especially for individuals with predisposing factors such as compromised lymphatic drainage or chronic skin conditions like stasis eczema, asteatotic eczema, atopic eczema, psoriasis, and intertrigo (5, 6). Notably, obesity has been identified as an independent risk factor for local complications of erysipelas, including bullae, abscesses, and necrosis, which may further increase morbidity and healthcare costs (7). As such, erysipelas remains a public health concern (8), particularly for populations at risk for severe complications and recurrence.

Despite advances in understanding the pathophysiology of erysipelas, challenges persist in diagnosis, treatment, and prevention. In China, the treatment of erysipelas poses unique challenges as most dermatology departments are outpatient-only and do not provide intravenous therapy, necessitating the transfer of care to emergency or surgical departments. In clinical practice, the effective diagnosis of erysipelas is often challenging, as its clinical manifestations may overlap with other skin and soft tissue infections (SSTIs), osteomyelitis, or even resemble conditions such as stasis dermatitis, necrotizing fasciitis, chronic allergic contact dermatitis, deep vein thrombosis (DVT) (9), and erysipelas-like erythema (ELE) in familial Mediterranean fever (FMF) (10), potentially leading to misdiagnosis or delays in appropriate treatment (11–13). Additionally, the management of erysipelas is challenged by emerging antimicrobial resistance, particularly methicillin-resistant *Staphylococcus aureus* (MRSA) (14), and a high likelihood of recurrence (3), which is further exacerbated in individuals with underlying diseases (15). Furthermore, recent studies have proposed gut microbiota dysbiosis as a novel contributor to disease susceptibility and recurrence, suggesting that microbiota modulation may offer new therapeutic opportunities (16). Given these diverse concerns, it is essential to develop an up-to-date, comprehensive understanding of erysipelas, encompassing its epidemiology, associated risk factors, and effective therapeutic approaches.

This study seeks to address these gaps by conducting a bibliometric analysis of erysipelas research. Bibliometric methods provide valuable insights into the research landscape, allowing for the identification of publication trends, key research areas, and collaborative networks (17). By utilizing a bibliometric approach, this study aims to reveal the underlying structure and evolution of erysipelas research, which can help inform clinical practice and direct future research efforts toward unresolved questions in the field. This approach is particularly valuable in an area like erysipelas, where multidisciplinary perspectives from dermatology, epidemiology, and infectious disease studies converge.

The present study utilizes data from the Web of Science Core Collection (WoSCC), specifically the Science Citation Index Expanded (SCI-EXPANDED) database, covering publications from 2000 to 2024. Through this analysis, we systematically examine publication volume, co-authorship patterns, geographic distribution, keyword trends, and institutional collaborations. These findings are visualized and analyzed using VOSviewer (18), bibliometrix (19), and CiteSpace (20) to map the knowledge structure and emerging trends within erysipelas research. The outcomes of this study are intended to guide future research directions and improve the clinical and preventive approaches to erysipelas management.

## 2 Materials and methods

### 2.1 Literature search and data collection

This bibliometric study focused on erysipelas, with data sourced from the Web of Science Core Collection (WoSCC), specifically the Science Citation Index Expanded (SCI-EXPANDED) database. WoSCC was selected due to its broad coverage and reliable classification of document types. The search was carried out using the following query: TS = (Erysipelas) AND TS = (“Infection” OR “Treatment” OR “Epidemiology” OR “Therapy” OR “Diagnosis” OR “Etiology” OR “Cause”). The date range for the search was set from January 1, 2000, to October 16, 2024, ensuring a wide range of relevant studies.

**Figure 1:**
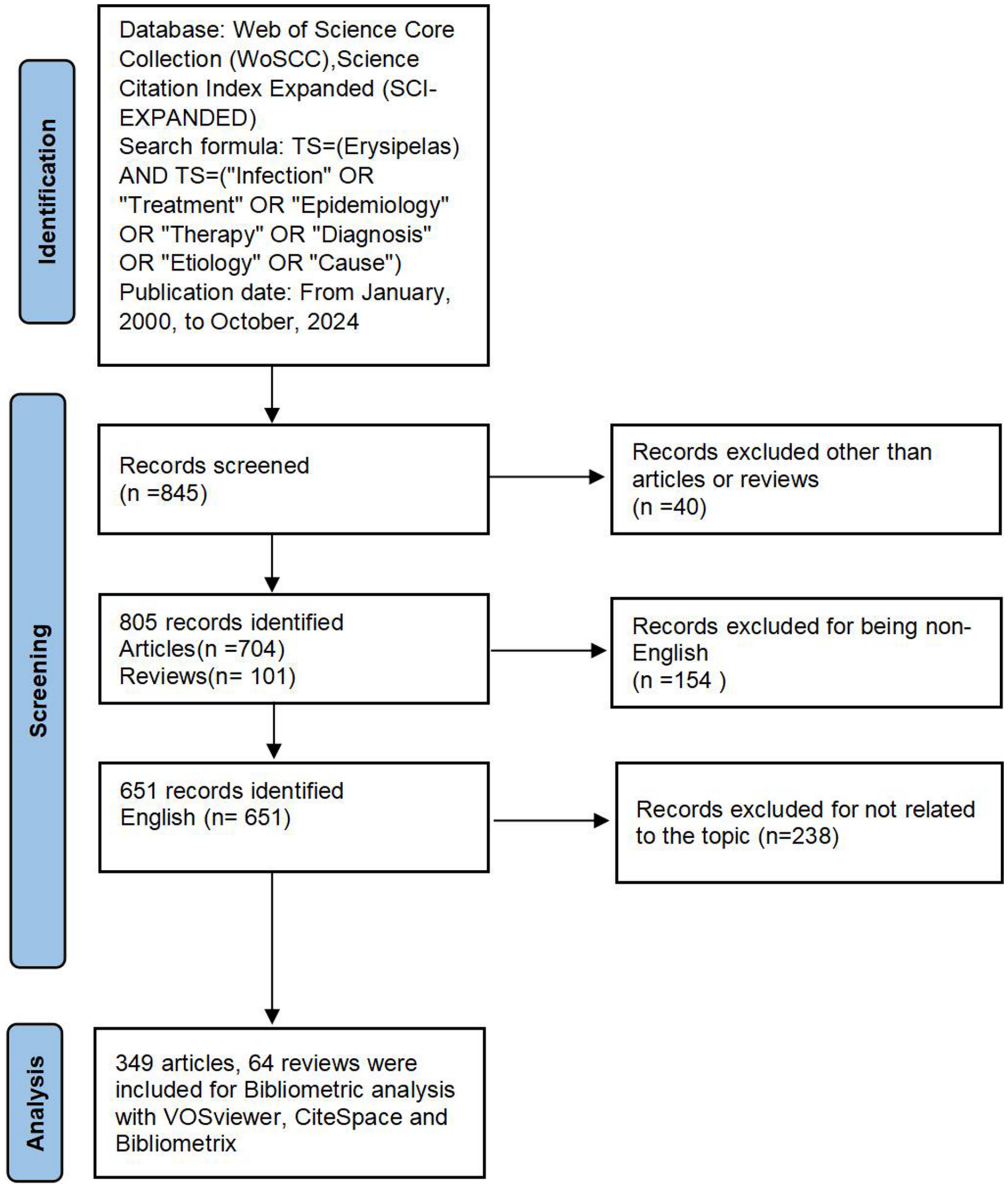
The flowchart of literature selection and bibliometric analysis in erysipelas.

Only articles and reviews published in English and reporting original research findings were included. All searches were performed October 16, 2024, to avoid discrepancies arising from potential database updates. Two reviewers independently assessed the retrieved publications for relevance to the study. After excluding non-English documents, irrelevant studies, and documents that were not classified as articles or reviews, 349 articles and 64 reviews were selected for final analysis, resulting in a total of 413 publications. The dataset was saved in plain text format (Figure 1).

### 2.2 Data analysis

For the analysis of the selected literature, various bibliometric tools were employed to examine research patterns and collaborations in erysipelas. VOSviewer (version 1.6.20) was used to visualize the co-authorship network, institutional affiliations, keyword co-occurrence, and density maps. The global network of international collaborations was mapped using Scimago Graphica, providing a clear, interpretable view of research cooperation across countries and regions. CiteSpace (version 6.2.R3) was employed for burst detection and dual-map overlays, enabling the identification of hotspots within erysipelas research. Furthermore, the bibliometrix package in R was used to create a Three Fields Plot and to analyze topic trends over time, providing deeper insights into evolving research themes.

In these visual representations, nodes signify different entities, such as authors, institutions, countries, or keywords, with the size of the node indicating its relative frequency or importance. The nodes and connecting lines are color-coded to distinguish between clusters or time periods, while the line thickness reflects the strength of relationships or collaborations.

## 3 Results

### 3.1 Publication volume and growth trends

**Figure 2:**
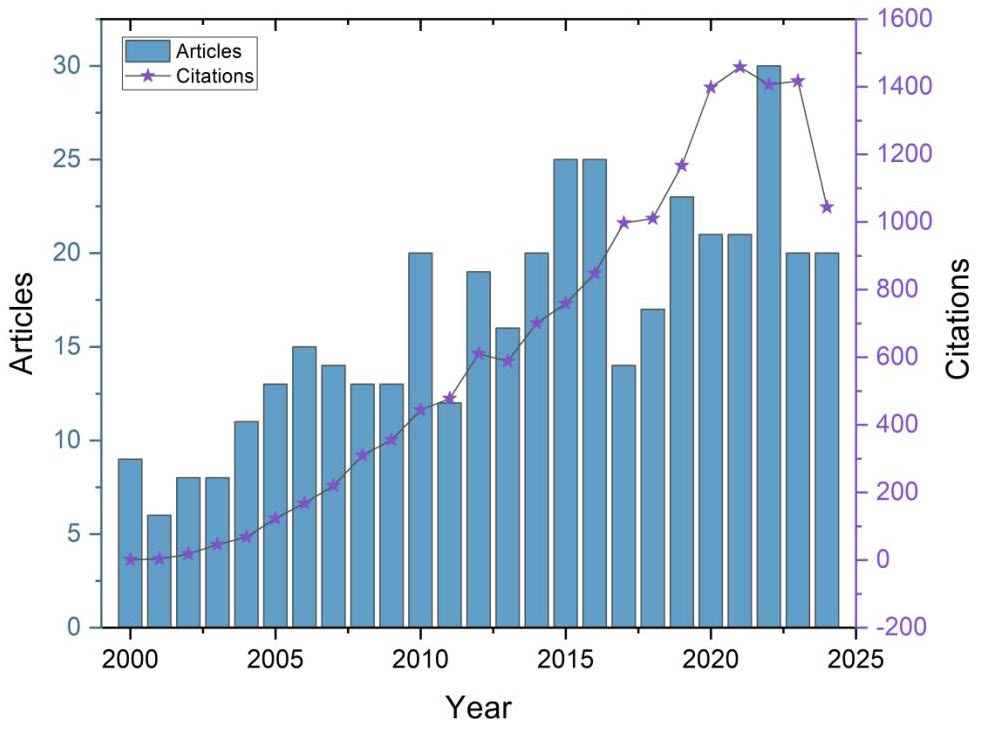
Annual trend of publications and citations in erysipelas research.

The annual publication trends related to erysipelas from 2000 to 2024 show a steady increase in research output, with the number of articles and citations rising significantly over the two-decade period (Figure 2). In 2000, there were only 9 published articles, while by 2022, the annual count reached a peak of 30 articles. This upward trend in article production highlights growing interest and research activity in the field, potentially driven by factors such as rising incidence rates, increasing antibiotic resistance, and the clinical complexity of recurrent infections.

The citation trends mirrored the increase in publications, with a sharp rise in citation numbers over time. In the early 2000s, the citation count was relatively low, starting with less than 10 citations in 2000. However, from 2010 onwards, the field saw an acceleration in citations, peaking at 1,458 citations in 2021. This increase suggests that not only has the quantity of research expanded, but its impact and relevance within the scientific community have also grown considerably. The decline in citations post-2021 may reflect the time lag typically associated with academic citations catching up to more recent publications.

### 3.2 Country/region analysis

**Figure 3:**
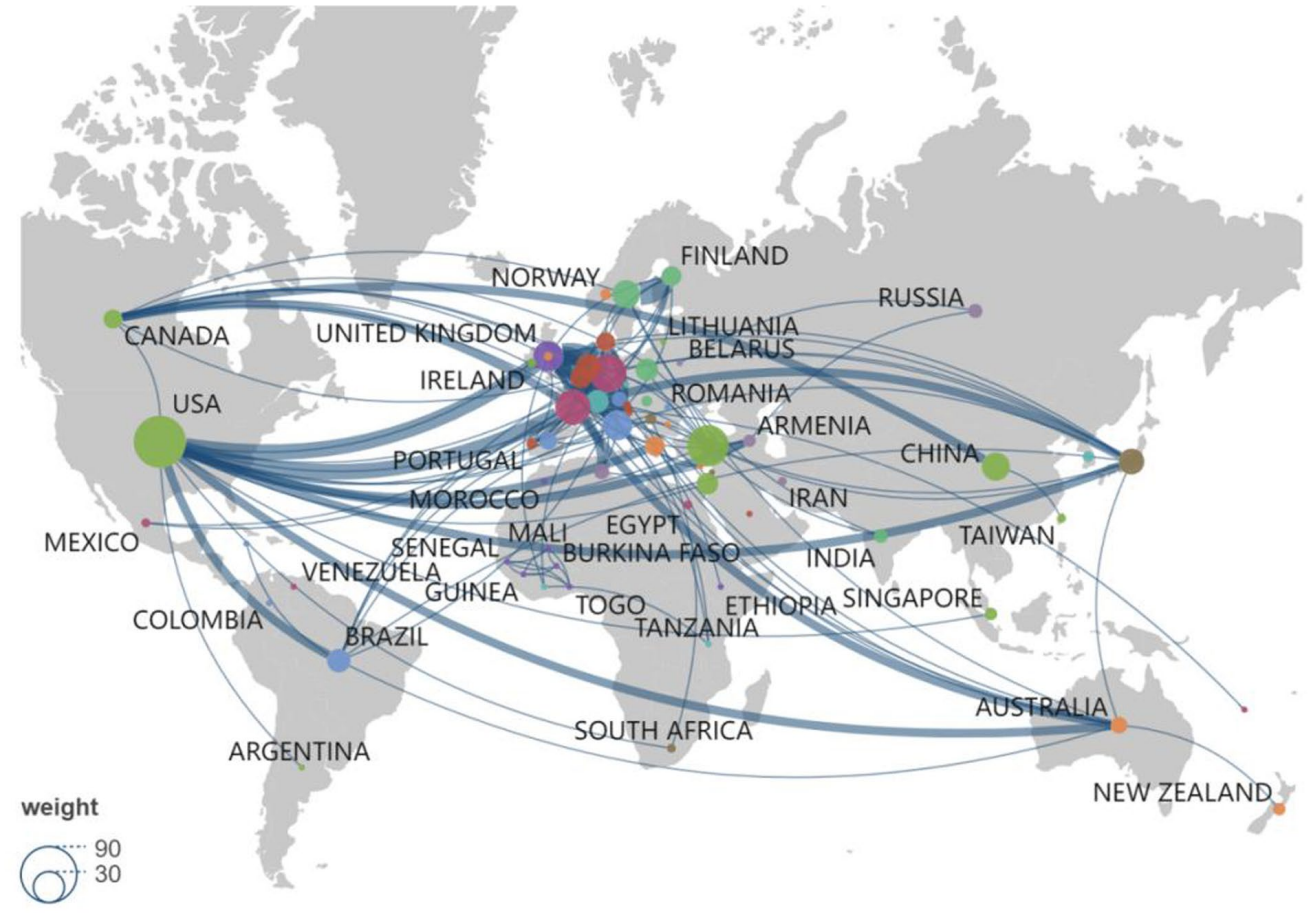
Global distribution and international collaboration network of erysipelas publications by country/region.

This analysis reveals notable geographic variations in erysipelas research impact. The United States leads with 69 publications and 2,739 citations, achieving a citation per article of 39.70. Turkey follows with 44 publications and a citation per article of 31.18. Despite fewer publications, the Netherlands stands out with a high citation per article of 70.5, indicating strong influence (Table 1). The collaboration network (Figure 3) demonstrates strong research partnerships in erysipelas research, with the United States and Europe as key hubs. The USA shows robust connections with the UK, Canada, and several other European countries, indicating significant collaboration. The UK similarly maintains strong ties with European nations like Germany and the Netherlands, fostering a dynamic research environment. Canada’s links with the USA,China and Europe reflect active joint research efforts. Emerging collaborations are also observed with countries like Japan, Brazil and Australia, suggesting growing global integration. Overall, the network map reveals concentrated collaboration primarily within the USA and Europe, with growing connections to Asia, Australia, and Brazil, which enrich the global research landscape.

**Table 1:** The top 10 countries/regions with the most significant contributions to erysipelas research.

Rank	Country/Region	Publications	Citations	Citation per article
1.00	USA	69.00	2739.00	39.70
2.00	Turkey	44.00	1372.00	31.18
3.00	Germany	36.00	1230.00	34.17
4.00	France	32.00	822.00	25.69
5.00	England	23.00	1514.00	65.83
6.00	Italy	23.00	1029.00	44.74
7.00	China	20.00	177.00	8.85
8.00	Sweden	20.00	670.00	33.50
9.00	Japan	17.00	272.00	16.00
10.00	Netherlands	16.00	1128.00	70.50

### 3.3 Institution analysis

**Figure 4:**
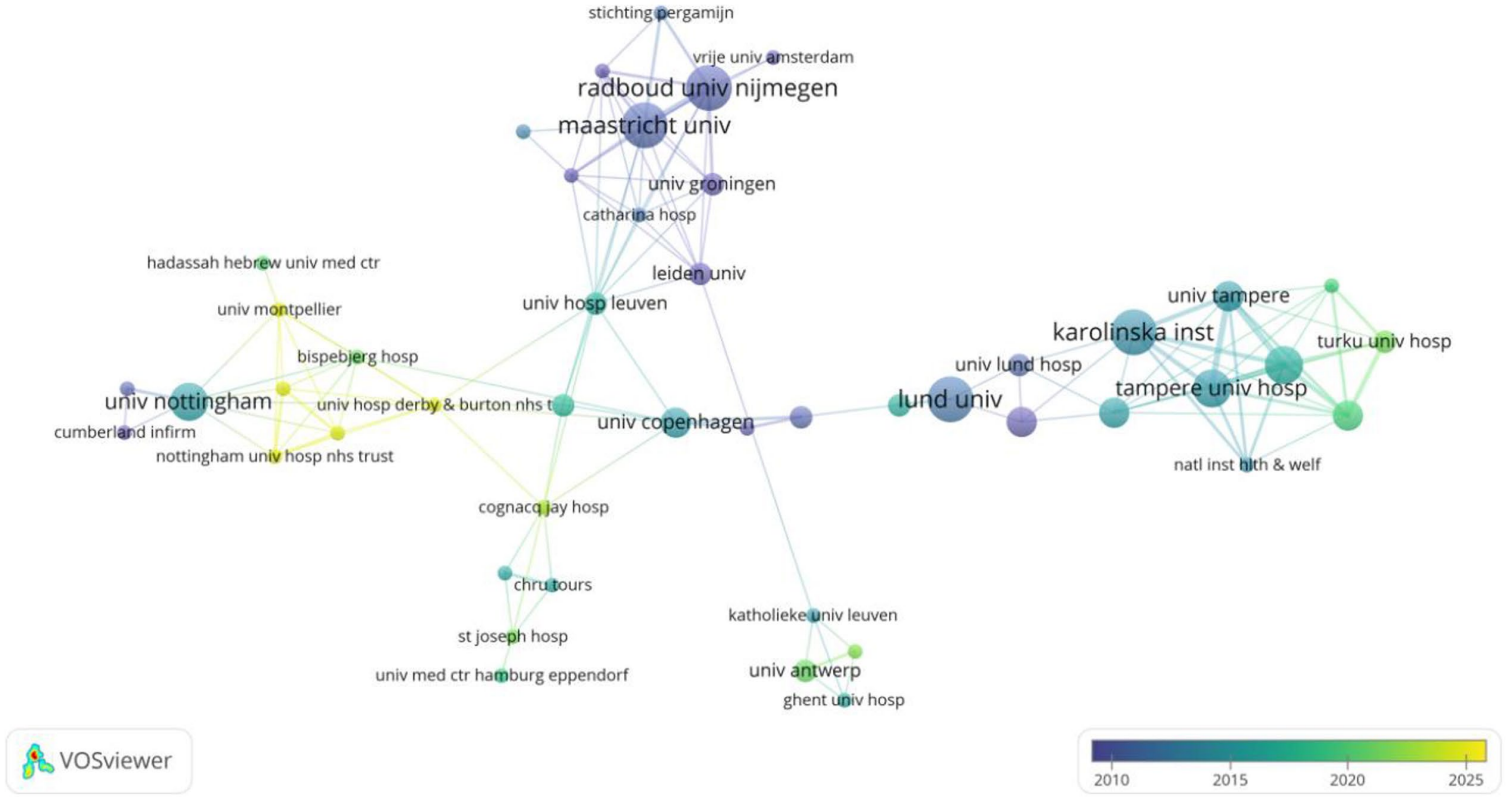
Co-authorship analysis of institutions in erysipelas.

The institutional analysis examines the contributions and collaborations of leading research organizations in erysipelas studies. Institutions such as Karolinska Institute, Lund University, Maastricht University, and Radboud University Nijmegen are top contributors, each with six publications. Radboud University Nijmegen leads in citation impact, with an average of 130.33 citations per article, reflecting the high influence of its research (Table 2). The collaborative network, centered primarily in Europe, shows strong connections between Dutch and Swedish institutions, particularly Radboud University Nijmegen, Maastricht University, Karolinska Institute, and Lund University. Evolving collaboration trends reveal that while established partnerships remain strong, newer collaborations are forming between institutions in Finland and the Netherlands, such as Tampere University Hospital and Turku University Hospital, expanding the scope of research (Figure 4).

**Table 2:** The top 10 institutions by publication volume in erysipelas research.

Rank	Organization	Country/Region	Publications	Citations	Citation per article
1	Radboud University Nijmegen	Netherlands	6	782	130.33
2	Maastricht University	Netherlands	6	749	124.83
3	Karolinska Institute	Sweden	6	216	36.00
4	Lund University	Sweden	6	177	29.50
5	Tampere University Hospital	Finland	5	245	49.00
6	University of Helsinki	Finland	5	154	30.80
7	University of Nottingham	England	5	89	17.80
8	Sahlgrenska University Hospital	Sweden	4	262	65.50
9	University of Tampere	Finland	4	154	38.50
10	Karolinska University Hospital	Sweden	4	149	37.25

### 3.4 Journal analysis

**Figure 5:**
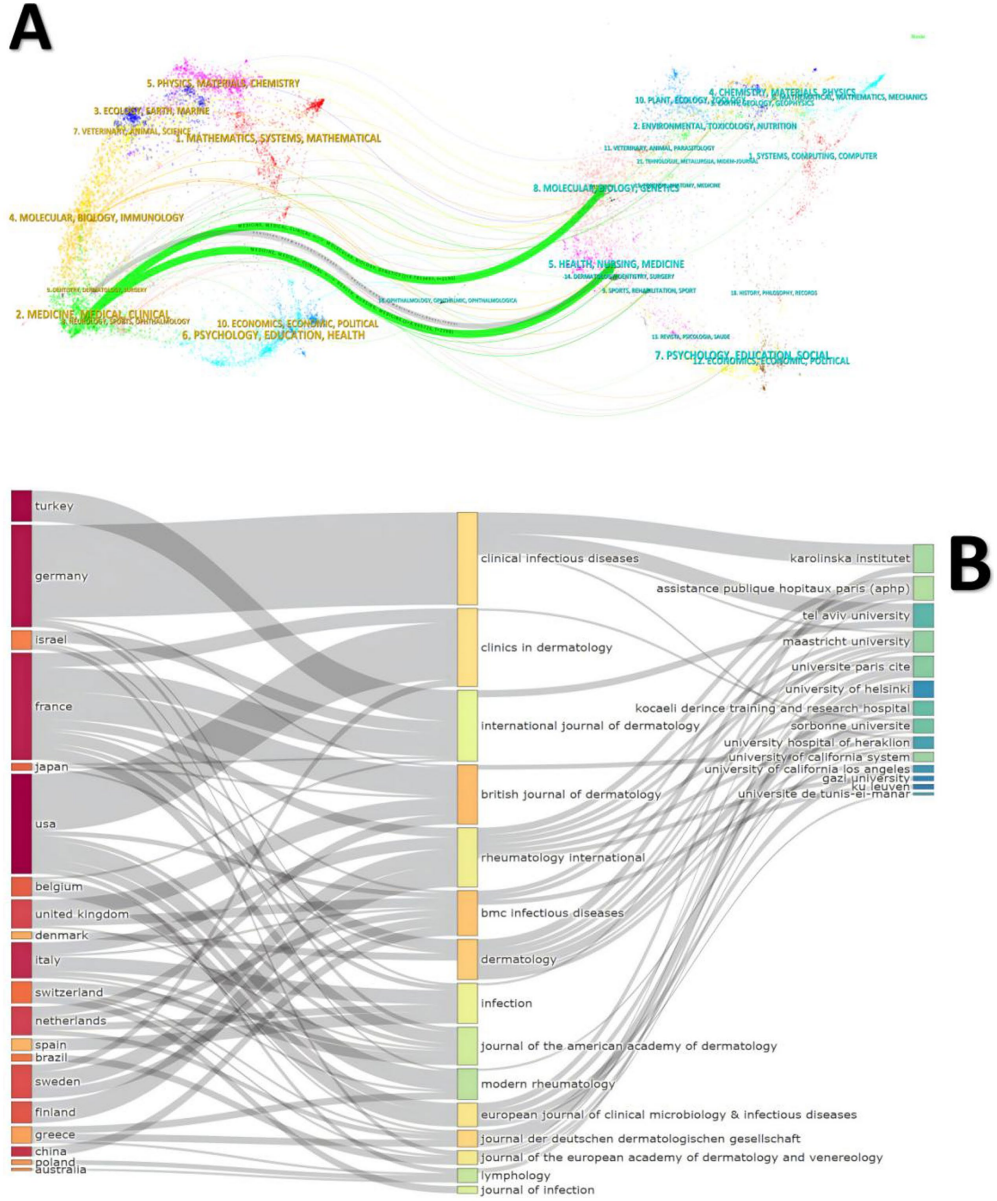
**(A)** Dual-map overlay of journals about erysipelas. **(B)** The three fields plot.

The dual-map overlay generated by Citespace (Figure 5A) and the three-field plot (Figure 5B) from Bibliometrix together offer a comprehensive perspective on citation patterns and key contributors in erysipelas research. The dual-map overlay illustrates that the primary citing fields, such as Medicine, Medical, Clinical, predominantly cited journals within the cited fields of Health, Nursing, Medicine and Molecular, Biology, Genetics. Among the top 10 journals with the highest publication volume (Table 3), the British Journal of Dermatology leads with 11 publications, 416 citations, and a high impact factor (IF 2023: 11). Other notable journals include BMC Infectious Diseases and Dermatology, each contributing 8 publications. These journals, through extensive international collaboration among the leading research institutions, serve as key platforms for advancing interdisciplinary studies on erysipelas.

**Table 3:** The top 10 journals with the highest number of publications in the field of erysipelas.

Rank	Journal	Publications	Citations	Citation per article	IF (2023)	JCR (2023)
1	British Journal of Dermatology	11	416	37.82	11	Q1
2	BMC Infectious Diseases	8	116	14.50	3.4	Q2
3	Dermatology	8	196	24.50	3	Q2
4	Journal der Deutschen Dermatologischen Gesellschaft	8	202	25.25	5.5	Q1
5	Clinical Infectious Diseases	7	228	32.57	8.2	Q1
6	Clinics in Dermatology	7	46	6.57	2.3	Q2
7	European Journal Of Clinical Microbiology & Infectious Diseases	7	266	38.00	3.7	Q2
8	Journal of the European Academy of Dermatology and Venereology	7	195	27.86	8.4	Q1
9	Rheumatology International	7	175	25.00	3.2	Q2
10	Infection	6	75	12.50	5.4	Q1

### 3.5 Reference analysis

**Figure 6:**
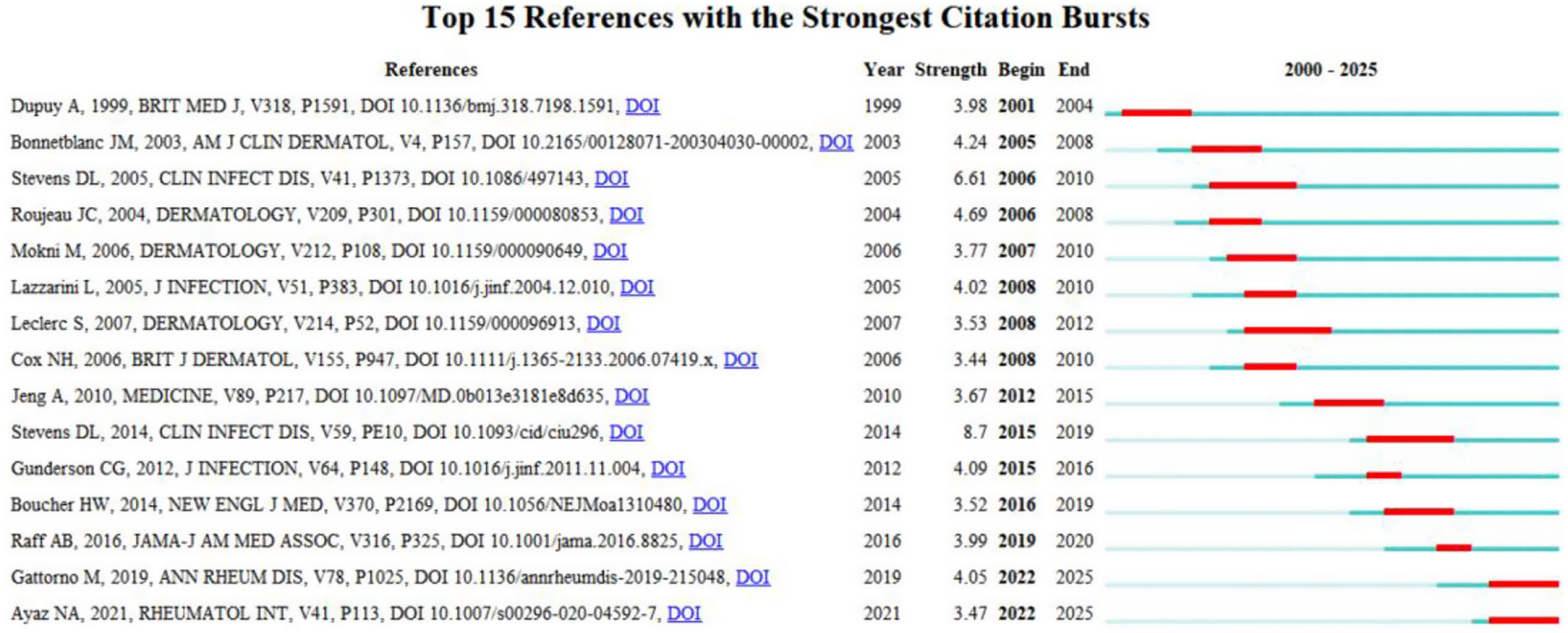
Top 15 references with the strongest citation bursts.

As shown in Figure 6, the top 15 references with the strongest citation bursts which was generated by Citespace highlights influential studies in erysipelas research. Among these, the guidelines by Stevens et al. (21, 22), with peak citation bursts of 8.7 (2015–2019) and 6.61 (2006–2010), have had a substantial impact on clinical approaches to erysipelas management. These guidelines underscore critical diagnostic and therapeutic strategies specific to erysipelas, with a particular focus on the challenges of antibiotic resistance in treatment protocols. Their work has established a foundational framework that continues to guide updates in clinical practice for effective erysipelas management within the broader context of SSTIs. Furthermore, Roujeau et al., with a citation burst of 4.69 (2006–2008), identified predisposing factors such as tinea pedis and onychomycosis, contributing valuable insights into preventive strategies (23). Recent studies by Gattorno et al. (burst value 4.05) and Ayaz et al. (burst value 3.47) both draw attention to Familial Mediterranean Fever as a potential cause of erysipelas-like erythema, underscoring the importance of differential diagnosis to accurately distinguish it from true erysipelas (24, 25).

### 3.6 Analysis of keywords and frontiers

**Figure 7:**
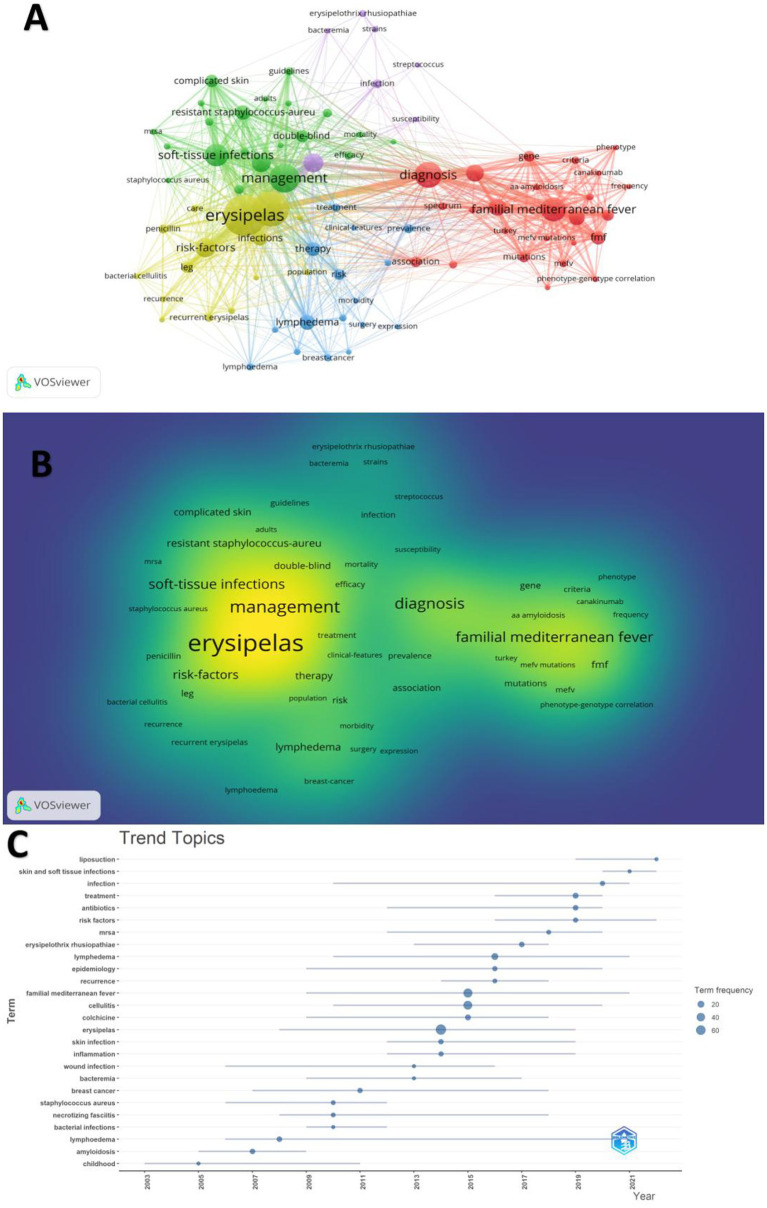
**(A)** Co-occurrence map of keywords. **(B)** Density map of keywords. **(C)** Trend topics.

A comprehensive keyword co-occurrence analysis was conducted using VOSviewer to highlight research hotspots (Figures 7A,B). In this study, 1,929 keywords were initially identified, with 84 keywords meeting the threshold criterion of six or more occurrences across the dataset. This selection yielded five primary clusters, each representing distinct research directions. The closest keywords in these five main clusters—represented by colors red, green, blue, yellow, and purple—are as follows: (1) familial Mediterranean fever, diagnosis, criteria, etc.; (2) management, soft-tissue infections, complicated skin conditions, etc.; (3) lymphedema, therapy, risk, etc.; (4) erysipelas, infections, risk factors, etc.; (5) bacteremia, strains, susceptibility, etc. Keyword frequency counts further revealed that Erysipelas (104), Cellulitis (83), Management (63), and Diagnosis (55) were the most frequently appeared terms, highlighting the dominant clinical themes. Familial Mediterranean Fever appeared in both full (49) and abbreviated (FMF, 22) forms, other notable terms included Soft-tissue infections (45), Risk factors (36), Skin (35), and Lymphedema (25).

An analysis of keyword trends further elucidates the evolving focal points within this research domain (Figure 7C). From 2000 to 2024, notable keywords included infection, epidemiology, familial Mediterranean fever, erysipelas, and lymphedema, reflecting dominant research themes over the years. In recent years (2020–2024), keywords such as liposuction and skin and soft tissue infections have emerged as significant areas of focus, representing current research priorities within the field.

## 4 Discussion

### 4.1 General information

The findings of this bibliometric analysis provide a comprehensive overview of the research landscape surrounding erysipelas, highlighting key trends, influential authors, and collaborative networks that shape the field. The growth in publication volume and citations over the past two decades suggests a sustained and increasing interest in erysipelas, likely driven by the clinical and public health implications associated with recurrent infections and the challenges posed by antibiotic resistance. This growth in scientific output reflects the pressing need for a deeper understanding of erysipelas’ etiology, risk factors, and effective treatment and prevention strategies.

The country-level analysis revealed that erysipelas research is predominantly concentrated in the United States and several European countries, with strong collaborative networks primarily involving North American and European institutions. The predominance of publications from Europe and America may stem from multiple factors, including regional disease prevalence and robust healthcare infrastructure, which enhance diagnostic and reporting rates. Additionally, historical leadership in medical research, coupled with greater access to funding and expertise, likely contributes to this publication trends. The high citation impact observed for studies from the Netherlands, despite a smaller publication volume, indicates the significant influence of Dutch research on erysipelas-related knowledge. Such concentrated regional collaborations suggest that certain high-impact research hubs have been instrumental in advancing the understanding and management of erysipelas, particularly through the exploration of antibiotic resistance patterns and innovative therapeutic approaches. Notably, emerging collaborations with countries like Japan, Brazil and Australia imply a shift towards more globalized research efforts, a promising development given the global burden of erysipelas and the potential for diverse epidemiological data to enrich understanding of its pathogenesis.

The institutional analysis underscores the central influence of key research organizations in defining the trajectory of erysipelas research. Beyond their notable scholarly output and significant academic influence, these institutions are integral to a robust collaborative framework, as depicted in Figure 4. The network visualization highlights intricate interconnections between institutions, fostering a tightly knit scholarly ecosystem. Notably, these partnerships extend beyond sporadic collaborations, instead forming a comprehensive and interdependent system that supports ongoing knowledge sharing and coordinated scientific efforts. Such cooperative frameworks are essential for advancing erysipelas research, particularly given the disease’s complexity, including challenges in diagnosis, recurring infections, and rising antimicrobial resistance. The stability and gradual expansion of this collaborative network provide a resilient platform for future inquiry, enabling the convergence of multidisciplinary expertise, accelerating the dissemination of findings, and stimulating innovation in both fundamental and clinical aspects of erysipelas research.

The journal analysis revealed that much of the seminal erysipelas research is published in high-impact clinical medicine journals, underscoring the multidisciplinary nature of this field, which spans dermatology, infectious disease, and general medical sciences. The British Journal of Dermatology, BMC Infectious Diseases, and Dermatology were identified as key publication venues, reflecting the clinical emphasis of erysipelas research on skin infections and therapeutic interventions. This distribution suggests that clinical outcomes and therapeutic strategies are core focuses within erysipelas studies, aligning with the field’s overarching goals of improving diagnostic accuracy and managing recurrent infections.

### 4.2 Hotspots and frontiers

In terms of risk factors, research has consistently emphasized the importance of skin health. Roujeau JC’s work from 2004 identified tinea pedis and other dermatological conditions as significant contributors to erysipelas risk, underscoring the need for preventive measures, particularly in individuals with a history of erysipelas or recurrent infections (23). Other studies align with this focus, indicating that breaks in skin integrity, including those due to wounds, chronic venous insufficiency, and lymphedema, are crucial risk factors for erysipelas (6, 26, 27). Furthermore, recent studies found that liposuction significantly reduces the incidence of erysipelas in patients with postmastectomy arm lymphedema, suggesting its effectiveness in minimizing infection recurrence (28, 29). These insights highlight the need for targeted preventive protocols and patient education in medical practices.

Differential diagnosis of erysipelas is another hotspot due to the overlap in clinical manifestations with other conditions that present with lower limb erythema and swelling, such as stasis dermatitis, necrotizing fasciitis, chronic allergic contact dermatitis, DVT (9) and ELE associated with FMF (10). Erysipelas typically presents with sudden onset of erythema, swelling, and tenderness, often accompanied by systemic symptoms such as fever and chills, which are distinguishing features (9, 11). However, traditional biomarkers like C-reactive protein (CRP) and white blood cell counts lack specificity for erysipelas, whereas elevated procalcitonin (PCT) levels have shown a high discriminatory value, correlating with infection severity and aiding in the differentiation from DVT (30). According to current clinical guidelines, duplex sonography plays an essential role in ruling out DVT in patients presenting with erysipelas-like symptoms (9). While it is a valuable tool for excluding DVT, its diagnostic accuracy is insufficient for identifying deeper soft tissue infections such as necrotizing fasciitis. In patients with atypical clinical features, extensive erythema, or systemic signs suggestive of severe infection, additional imaging modalities, particularly computed tomography (CT) or magnetic resonance imaging (MRI), are recommended to ensure accurate differential diagnosis (31). In cases mimicking erysipelas, such as ELE in FMF patients, histopathological examination reveals perivascular lymphocytic infiltration without vasculitis, alongside recurrent erythematous plaques that resolve within 48–72 h, often recurring at the same anatomical site (32).

The advent of omics technologies has added a new dimension to erysipelas research, particularly with regard to understanding the microbiome’s role in skin health and systemic inflammation. High-throughput sequencing techniques have allowed researchers to delve into the complex relationship between the gut microbiota and skin inflammation, highlighting dysbiosis as a possible contributor to erysipelas susceptibility (33). Keywords like “gut microbiota,” “inflammation,” and “dysbiosis” have become prominent in recent studies, reflecting the field’s growing interest in microbiota-targeted therapies for inflammatory skin conditions, including erysipelas. Bao et al. demonstrated that imbalances in gut microbiota could exacerbate inflammation, suggesting a potential role for microbiota modulation in the management of recurrent erysipelas (16). These findings underscore an emerging area in erysipelas research, where the gut-skin axis may be targeted to improve patient outcomes.

Building upon the understanding of risk factors, diagnosis, and emerging therapeutic targets, the cornerstone of erysipelas management remains timely and appropriate antimicrobial therapy. Penicillin G continues to be the first-line treatment due to its proven efficacy against *Streptococcus pyogenes*, the predominant causative agent (34). Alternative agents, such as amoxicillin and macrolides, are used in cases of penicillin allergy, though the efficacy of these treatments remains well-established for streptococcal infections (35, 36). However, due to potential differences in allergic determinants among penicillin variants and cephalosporins, specific IgE testing is recommended in cases of suspected allergy to ensure appropriate antibiotic selection, especially considering rare but severe hypersensitivity reactions even after long-term penicillin tolerance (37). Additionally, Bernard et al. conducted a comparison between oral pristinamycin and standard penicillin therapy, demonstrating that pristinamycin can effectively treat erysipelas in outpatient settings, providing a practical alternative that reduces hospital resource use (38).

Meanwhile, the emergence of antibiotic resistance, particularly among non-streptococcal pathogens, complicates management, especially when mixed infections are suspected. In cases where *Staphylococcus aureus* is involved, which can occur in bullous or hemorrhagic forms of erysipelas, broader-spectrum antibiotics or combination therapy may be required (14). In addition to antimicrobial therapy, supportive measures are essential components of comprehensive erysipelas management. Elevation of the affected limb and the use of compression therapy are recommended to reduce edema, improve lymphatic drainage, and lower the risk of recurrence, particularly in patients with underlying lymphedema (39). Furthermore, based on our clinical experience, appropriate pain management, meticulous skin care, and encouragement of physical activity are crucial for optimizing clinical outcomes and facilitating recovery.

### 4.3 Limitations

This study has several limitations. First, it relies solely on data from the Web of Science Core Collection, excluding other databases like PubMed or Scopus, which may result in the omission of relevant studies and limit the comprehensiveness of the analysis. Second, only English-language publications were included, introducing a potential language bias and underrepresenting research from non-English-speaking regions. Third, citation counts were used as a proxy for research impact, which may not accurately reflect clinical relevance or quality, and recent publications may be underrepresented due to the time required to accumulate citations.

## 5 Conclusion

This bibliometric analysis highlights the growing global interest in erysipelas research from 2000 to 2024, emphasizing advancements in understanding risk factors, improving diagnostic methods, and exploring novel therapeutic approaches. Despite significant contributions from leading countries and institutions, challenges such as antibiotic resistance, recurrence, and differential diagnosis remain critical areas for improvement. The findings underscore the importance of multidisciplinary collaboration and innovative strategies to address these challenges, providing a roadmap for future research to enhance the diagnosis, treatment, and prevention of erysipelas.

## Data Availability

The original contributions presented in the study are included in the article/supplementary material, further inquiries can be directed to the corresponding author.
